# Assessment and quantification of ovarian reserve on the basis of machine learning models

**DOI:** 10.3389/fendo.2023.1087429

**Published:** 2023-03-15

**Authors:** Ting Ding, Wu Ren, Tian Wang, Yun Han, Wenqing Ma, Man Wang, Fangfang Fu, Yan Li, Shixuan Wang

**Affiliations:** Department of Obstetrics and Gynecology, Tongji Hospital, Tongji Medical College, Huazhong University of Science and Technology, Wuhan, Hubei, China

**Keywords:** ovarian aging, ovarian reserve, machine learning, quantification, light gradient boosting machine

## Abstract

**Background:**

Early detection of ovarian aging is of huge importance, although no ideal marker or acknowledged evaluation system exists. The purpose of this study was to develop a better prediction model to assess and quantify ovarian reserve using machine learning methods.

**Methods:**

This is a multicenter, nationwide population-based study including a total of 1,020 healthy women. For these healthy women, their ovarian reserve was quantified in the form of ovarian age, which was assumed equal to their chronological age, and least absolute shrinkage and selection operator (LASSO) regression was used to select features to construct models. Seven machine learning methods, namely artificial neural network (ANN), support vector machine (SVM), generalized linear model (GLM), K-nearest neighbors regression (KNN), gradient boosting decision tree (GBDT), extreme gradient boosting (XGBoost), and light gradient boosting machine (LightGBM) were applied to construct prediction models separately. Pearson’s correlation coefficient (PCC), mean absolute error (MAE), and mean squared error (MSE) were used to compare the efficiency and stability of these models.

**Results:**

Anti-Müllerian hormone (AMH) and antral follicle count (AFC) were detected to have the highest absolute PCC values of 0.45 and 0.43 with age and held similar age distribution curves. The LightGBM model was thought to be the most suitable model for ovarian age after ranking analysis, combining PCC, MAE, and MSE values. The LightGBM model obtained PCC values of 0.82, 0.56, and 0.70 for the training set, the test set, and the entire dataset, respectively. The LightGBM method still held the lowest MAE and cross-validated MSE values. Further, in two different age groups (20–35 and >35 years), the LightGBM model also obtained the lowest MAE value of 2.88 for women between the ages of 20 and 35 years and the second lowest MAE value of 5.12 for women over the age of 35 years.

**Conclusion:**

Machine learning methods combining multi-features were reliable in assessing and quantifying ovarian reserve, and the LightGBM method turned out to be the approach with the best result, especially in the child-bearing age group of 20 to 35 years.

## Introduction

Ovarian reserve represents the number of oocytes remaining in the ovary; both the number and quality of oocytes impact reproductive potential and aging (1, 2). Ovarian aging is due to a variety of causative factors, such as chromosomal, genetic, mitochondrial, and cytoplasmic changes in oocyte quantity and quality (3–8). Evaluation of present ovarian reserve and ovarian aging degree could offer helpful advice for women regarding evaluating their reproductive potential and preventing early menopause or related disorders because few treatments are effective in preventing ovarian aging.

So far, the most classical and commonly used evaluation system for ovarian aging is the Stages of Reproductive Aging Workshop criteria (STRAW+10), which is widely considered the gold standard for characterizing reproductive aging through menopause. STRAW classified the stages of a woman’s adult life into three general categories: reproductive, menopausal transition, and postmenopause. However, the STRAW staging approach lacks specific diagnostic criteria for evaluating ovarian reserve, and the assessment system is too generalized to reliably assess each individual’s ovarian aging degree. In addition, the current evaluation of ovarian reserve can draw on clinical indicators, such as biochemical tests and ultrasound imaging of the ovaries (2). Biochemical tests include follicle-stimulating hormone (FSH), estradiol (E2), or inhibin B in early-follicular-phase, cycle-day-independent anti-Müllerian hormone (AMH), and provocative tests, while ultrasonographic measures include antral follicle count (AFC) and ovarian volume. Among these indicators, AMH is regarded as the most sensitive and reliable marker of ovarian reserve because it is independent of the menstrual cycle and tends to decline before FSH rises (9). However, several studies have reported the limited use of these markers. In reproductive-aged women without a history of infertility, markers of lower ovarian reserve were found to be unrelated to reduced fertility, and in women with a history of one to two previous miscarriages, AMH levels were found to be unrelated to clinical pregnancy loss (10, 11). These findings highlight the limitations of these single markers.

Machine learning holds considerable advantages for analyzing and integrating large amounts of medical data (12, 13). Machine learning can fully account for the interactions between characteristics and incorporate new data to update models, in contrast to traditional statistical analysis approaches, which rely on a preset equation (14). In the realm of assisted reproduction, machine learning methods have previously been applied to evaluate and predict pregnancy rates (15–17). Researchers also have attempted to construct regression models to assess ovarian reserve by integrating single biochemical and ultrasound markers (18–21). However, more machine learning methods should be utilized to determine a suitable evaluation model. The main aim of this study is to develop a more accurate machine learning model to estimate and quantify ovarian reserve in terms of predicting reproductive possibility and time to menopause.

## Method

### Population selection

This is a multicenter, nationwide population-based study. The participants were recruited from seven centers in six different cities of China, including the city of Shenyang (northern China), Foshan (southern China), Chengdu (western China), Zhengzhou, Yichang, and Wuhan (central China). From October 2011 to December 2014, a total of 2,055 women, aged 20 to 55, were recruited through advertisements. Of the initial recruits, 1,020 women met the following strict inclusion criteria for the healthy population used for modeling: 1) regular menstrual cycles between 21 and 35 days for women <40 years old having regular menstrual cycles and for women >40 not required to have regular menstrual cycles, considering that they may be in normal perimenopause or menopause; 2) no hormone use in the past 6 months; 3) no history of radiotherapy or chemotherapy; 4) no history of hysterectomy, oophorectomy, or any other type of ovarian surgery; 5) no ovarian cysts or ovarian tumors, as confirmed by ultrasound; and 6) no known chronic, systemic, metabolic, or endocrine diseases such as hyperandrogenism or hyperprolactinemia.

All volunteers were interviewed one-on-one using prepared questionnaires that included questions about their demographic, geographic, and reproductive characteristics. The participants were physically examined and received free hormone and ultrasound testing. The study was approved by the Tongji Ethics Committee, and written informed consent was obtained from each woman for the anonymous use of clinical data for statistical evaluation and research purposes.

### Blood sample collection

All blood samples were taken from the participants’ antecubital vein between 7:00 AM and 11:00 AM, after a 12 h overnight fast, on days 2 to 5 of a spontaneous menstrual cycle or any day if amenorrhea had lasted more than 3 months in those aged over 40 years. The samples were then centrifuged using standard conditions within 2 h of venipuncture. After centrifugation, serums were obtained, aliquoted, transported to the central laboratory, and stored at −80°C for no more than 2 weeks until the assays were performed. To avoid the potential bias produced by differences between laboratory test results, we chose the gynecologic endocrine laboratory of Tongji Hospital as the central laboratory; all serums were transported to the central laboratory using dry ice within 48 h of collection, and all serum hormones were tested in the central laboratory.

### Hormone detection

Serum concentrations of AMH at the time of recruitment were measured using the AMH Gen II ELISA kit (Beckman Coulter, Inc., Brea, CA, USA) and Ultra-Sensitive AMH ELISA assays (AL-105, Ansh Labs, Webster, TX, USA). Two commercial assays and the mean value were decided as the final AMH level, and all serum AMH measurements were performed in the same laboratory using the above kits. The controls were used at two concentrations to monitor the accuracy of the assay. The intra- and interassay coefficients of variation (CVs) were 3.6% and 4.5%, respectively. The lowest amount of AMH that could be detected with a 95% probability in a sample was 0.08 ng/ml for Gen II ELISA and 0.04 ng/ml for Ansh Labs; therefore, we replaced all values recorded as <min (undetectable) with a value of 0.08 or 0.04 ng/ml for the purpose of this analysis. Serum FSH, luteinizing hormone (LH), E2, testosterone (T), prolactin (PRL), and progesterone (PRG) levels were measured using a chemiluminescence-based immunometric assay on an ADVIA Centaur immunoassay system (Siemens Healthcare Diagnostics Inc., Tarrytown, NY, USA). All the serum hormone levels were measured in the same laboratory using the same kit. The intra- and interassay coefficients of variation were all <15%. Due to missing values, the three hormones—T, PRL, and PRG—were not included in the analysis.

### Ultrasound examination

A transvaginal ultrasound scan of the ovaries was performed to determine the AFC. This ultrasound examination was performed at the seven centers. All participating research institutes were modernized large comprehensive hospitals and received our regular supervision and verification. The unified standard for this examination was formulated in the beginning, and all ultrasound doctors were strictly trained to test AFCs according to the same standard. In this study, the AFC was defined as the total number of visible round or oval structures with diameters of 2 to 10 mm in both ovaries. All ultrasound examinations were performed on days 2 to 5 of a spontaneous menstrual cycle or in the follicular phase for non-menstruating women. None of the eligible participants had follicles larger than 10 mm. No significant differences were found between each center. The intra-analysis coefficient of variation for the follicle diameter measurements was <5%, and the lower limit of detection was 0.1 mm.

### Establishment and assessment of models

In this study, ovarian reserve was quantified in the form of ovarian age for healthy women, and ovarian age was regarded as equal to their chronological age. The least absolute shrinkage and selection operator (LASSO) regression was used for data regularization and feature selection (22). With the use of seven features (AMH, body mass index (BMI), Inhibin B, FSH, E2, LH, and AFC), quantifying work was performed. As for the construction of prediction models, seven different machine learning algorithms were used, namely artificial neural network (ANN), support vector machine (SVM), generalized linear model (GLM), K-nearest neighbors regression (KNN), gradient boosting decision tree (GBDT), extreme gradient boosting (XGBoost), and light gradient boosting machine (LightGBM), in which their chronological age was regarded as ovarian age in healthy women who were supposed to have a normal ovarian function (23–29). All the above-mentioned models were trained and tested on a partitioned 50/50 percentage split of the dataset by stratified random sampling. Parameter tuning was based on the grid search method and 10-fold cross-validation in training the dataset (30). The parameters of the machine learning models are listed in **Table S1**. For model assessment, Pearson’s correlation coefficient (PCC) and mean absolute error (MAE) values were applied to indicate how well a model explains the variation in the dependent variables. The mean squared error (MSE) value was calculated to measure the stability of the model. All machine learning techniques were programmed in R language (version 3.6.3) using packages including neuralnet, e1071, kknn, gbm, xgboost, and lightgbm.

## Results

**Figure 1:**
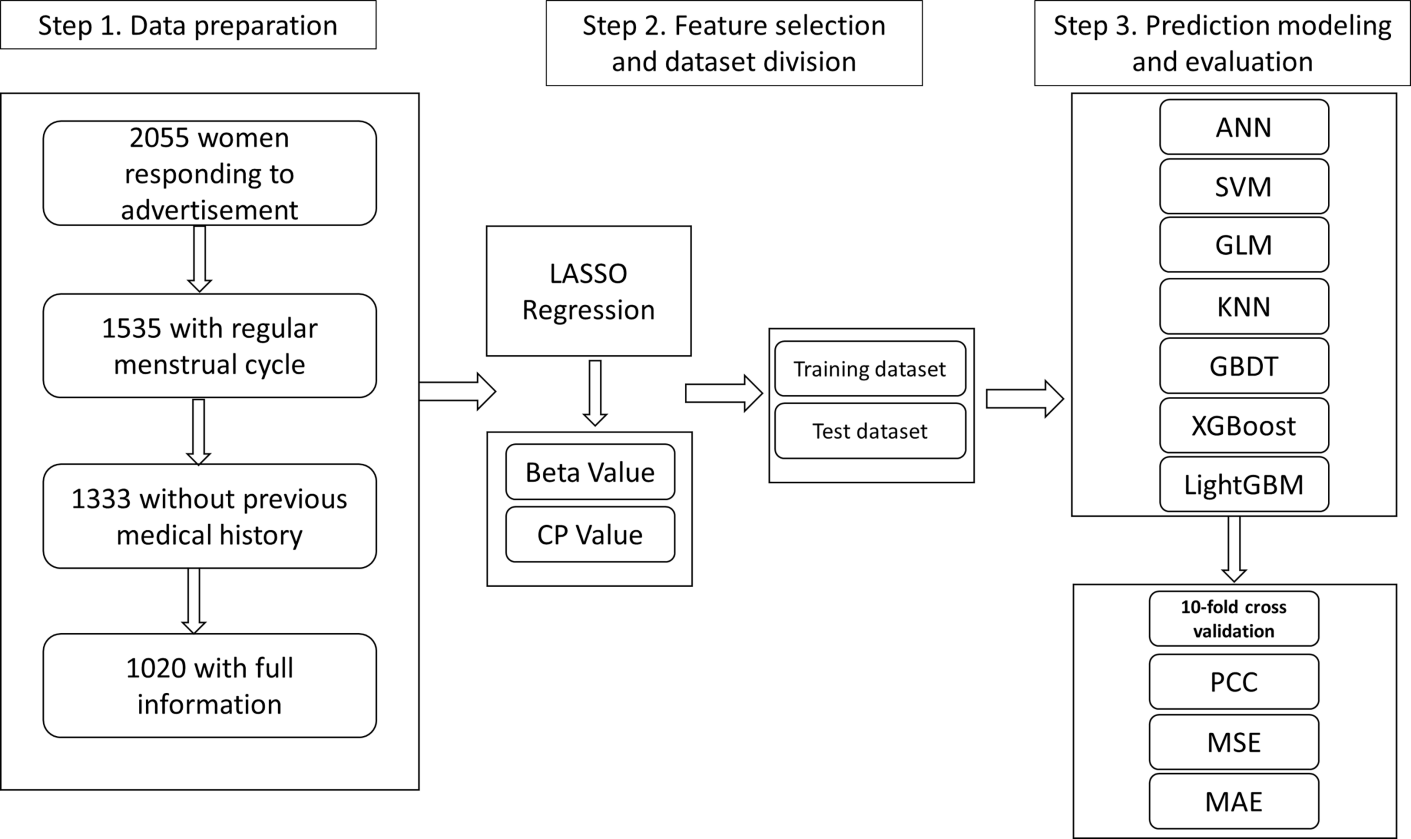
The flowchart of the study design.

**Figure 2:**
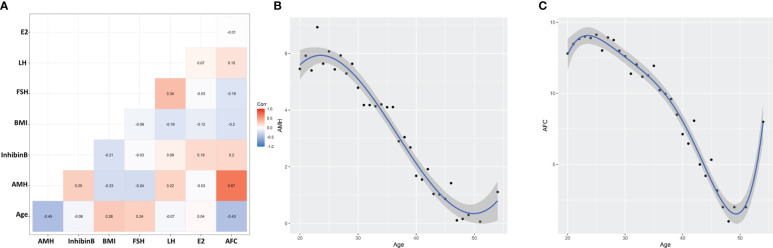
Correlation and distribution between features and age. **(A)** Pearson’s correlation coefficients between age and different features. **(B)** Age-specific anti-Müllerian hormone (AMH) value distribution and fitting curve. **(C)** Age-specific antral follicle count (AFC) value distribution and fitting curve.

From October 2011 to December 2014, a total of 2,055 women, aged from 18 to 55, were recruited through advertisements. According to exclusion and inclusion criteria, a total of 1,020 women from seven centers were enrolled and analyzed (**Figure 1**). **Table 1** summarizes the statistics of included women for age, AMH value, Inhibin B, BMI value, FSH, LH, E2, and AFC value. **Figure 2A** shows PCC between age and AMH (−0.45), Inhibin B (−0.08), BMI (0.28), FSH (0.24), LH (−0.07), E2 (0.04), and AFC (−0.43), from which AMH and AFC had the highest absolute value. In **Figures 2B, C**, the distribution curves are depicted for AMH and AFC within specific ages, which are similar to each other.

**Table 1:** Description of the features and centers from which the data were obtained.

Center	Number	Age (year)	AMH (ng/ml)	Inhibin B (pg/ml)	BMI	FSH (mIU/ml)	E2 (pmol/L)	LH (mIU/ml)	AFC
Chengdu	191	31.69 ± 5.20	3.79 ± 2.64	93.02 ± 31.95	21.52 ± 2.89	7.00 ± 1.87	53.07 ± 33.37	3.77 ± 1.92	10.68 ± 4.64
Foshan	246	31.40 ± 4.68	5.32 ± 3.34	95.56 ± 39.68	20.65 ± 2.72	8.00 ± 2.02	40.81 ± 20.19	4.81 ± 2.27	12.60 ± 2.62
SFY	13	33.19 ± 6.94	3.76 ± 4.00	83.16 ± 38.77	21.16 ± 4.06	8.42 ± 3.34	40.54 ± 20.91	4.33 ± 1.07	9.77 ± 5.33
Tongji	302	30.45 ± 5.54	4.89 ± 3.11	80.25 ± 29.55	21.30 ± 2.83	6.94 ± 2.81	41.85 ± 18.94	4.55 ± 2.71	13.32 ± 5.19
Shenyang	95	28.29 ± 7.74	5.72 ± 3.79	80.79 ± 38.13	20.58 ± 2.05	5.99 ± 3.23	45.06 ± 25.80	4.43 ± 2.04	11.68 ± 5.52
Yichang	15	33.17 ± 5.95	4.68 ± 3.35	76.39 ± 20.59	22.08 ± 3.11	7.40 ± 1.13	63.98 ± 25.18	4.40 ± 3.36	15.87 ± 6.45
Zhengzhou	158	33.32 ± 6.92	3.34 ± 2.73	78.62 ± 35.33	22.90 ± 3.26	7.67 ± 4.73	51.62 ± 46.89	5.35 ± 4.30	9.34 ± 4.96

AMH, anti-Müllerian hormone; BMI, body mass index; FSH, follicle-stimulating hormone; E2, estradiol; LH, luteinizing hormone; AFC, antral follicle count.

Holding the assumption that ovarian age was equal to chronological age in healthy women, we performed LASSO regression analysis on the total data to select those features suitable for constructing the models (**Figure S1**). Finally, these seven features were all left with the lowest CP value for the follow-up study (**Table S2**). We randomly chose half of the datasets as training data to make the prediction and half as test data, and we then checked the results on different datasets.

**Figure 3:**
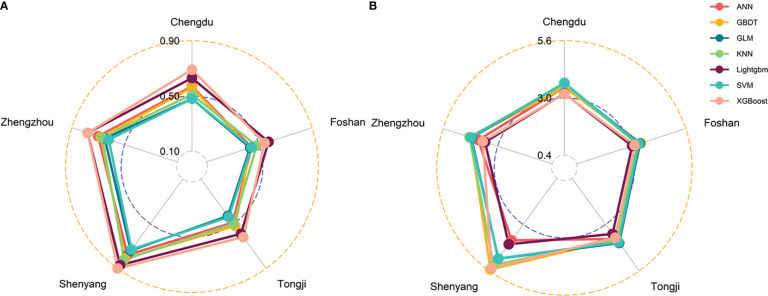
Radar plot showing correlation values **(A)** and mean absolute prediction errors (MAE) values **(B)** for the five datasets using the seven different prediction models.

The results of the prediction analyses were compared in terms of PCC and MAE values for the seven machine learning models (ANN, SVM, GLM, KNN, GBDT, XGBoost, and LightGBM) (**Table 2**). The table shows the PCC values for the training dataset, the test dataset, and the entire dataset, as well as the MAE values for the entire dataset. Focusing on the PCC values, it can be observed that the XGBoost, LightGBM, and ANN method had better performance. While PCC just describes the correlation trend, the MAE value represents the detailed difference, which reflects the prediction bias. The LightGBM model had the lowest MAE value for all the data of 3.41 years. As there were five datasets with more than 90 women, the seven models were also tested in the datasets of Chengdu, Foshan, Tongji, Shenyang, and Zhengzhou. The XGBoost and LightGBM models also obtained the highest PCC value in all center-based datasets. While the XGBoost model had the highest PCC value on the Shenyang dataset, at 0.90, the GLM model had the lowest value on the Foshan and Tongji datasets, at just 0.43 (**Figure 3A**). As for the MAE value, the GBDT model had the highest MAE value on the Shenyang dataset, at 5.52 years, and the LightGBM model had the lowest value on the Foshan dataset, at just 3.05 years (**Figure 3B**).

**Table 2:** Summary of prediction analyses for the training dataset (correlation value), the test dataset (correlation value), and the entire dataset (correlation value and mean absolute prediction errors value).

	Train	Test	Total	MAE*
ANN	0.68	0.56	0.62	3.68
SVM	0.57	0.55	0.56	3.91
GLM	0.61	0.51	0.56	3.93
KNN	0.58	0.62	0.60	3.87
GBDT	0.58	0.63	0.61	3.90
XGBoost	0.80	0.62	0.71	3.64
LightGBM	0.82	0.56	0.70	3.41

ANN, artificial neural network; SVM, support vector machine; GLM, generalized linear model; KNN, K-nearest neighbors regression; GBDT, gradient boosting decision tree; XGBoost, extreme gradient boosting; LightGBM, light gradient boosting machine.

*Mean absolute prediction errors for the entire dataset.

**Figure 4:**
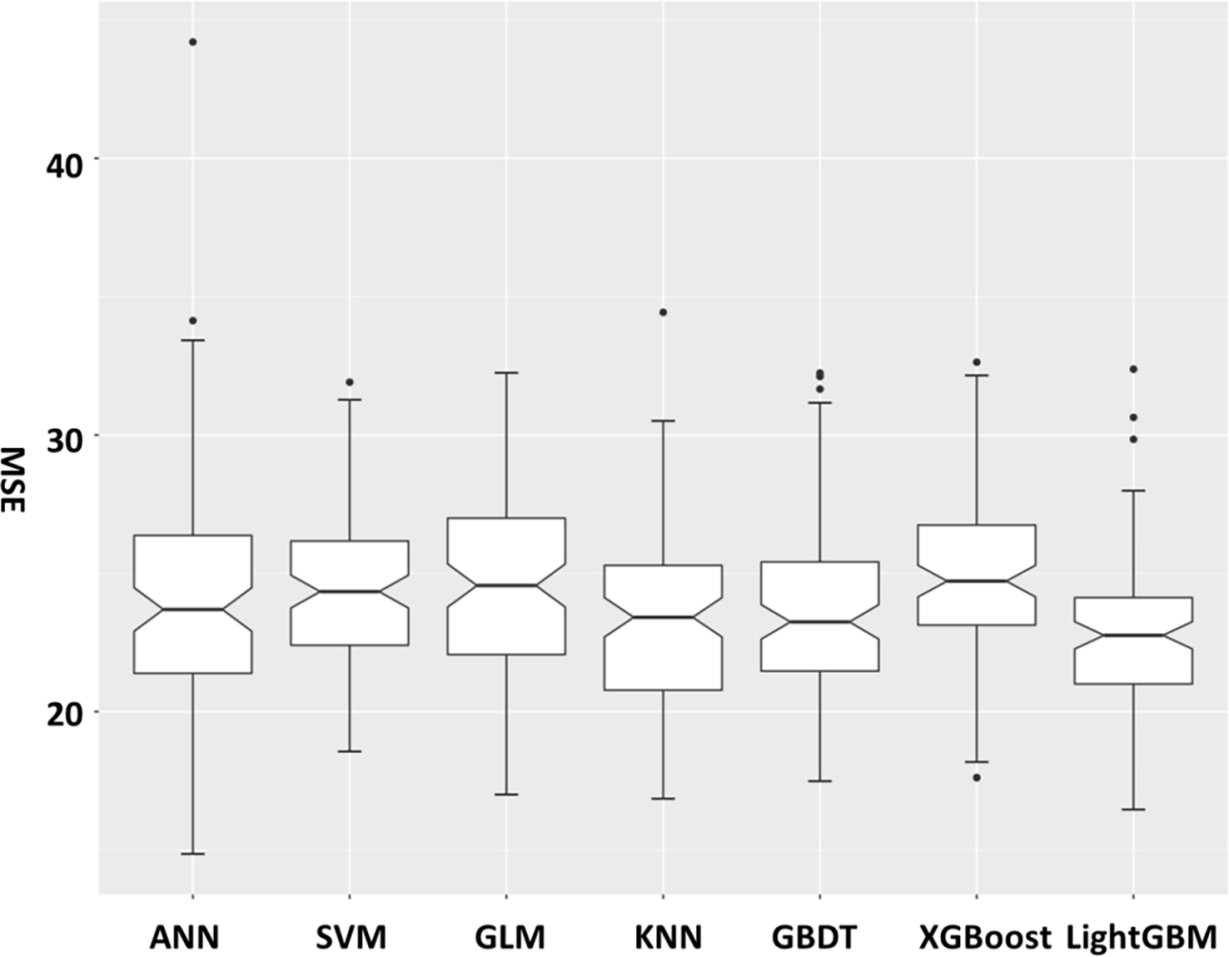
Mean squared error (MSE) after 10-fold cross-validation for the seven methods.

Further, we cross-validated the models using the 10-fold method in which we randomly chose 90% of the entire dataset as the training dataset and 10% of the data as the test dataset. We iterated the method 100 times and obtained a mean MSE value. **Figure 4** shows the MSE value broken down for the seven different methods. The lowest mean MSE was gained for the LightGBM technique, which showed the stability of this method.

In order to evaluate the performance of the models and select the most suitable one, we combined the three indexes of PCC value, MAE value, and MSE value. The models that ranked in the top three under each index were left. As shown in **Table 3**, the LightGBM model was the only one that ranked in the top three in all the lists. Though the PCC value of XGBoost was a little higher than that in the LightGBM model, the MSE and MAE values were much better in the LightGBM model.

**Table 3:** Models ranked top three under different evaluation indexes.

PCC	MAE	MSE
XGBoost	LightGBM	LightGBM
LightGBM	XGBoost	GBDT
ANN	ANN	KNN

PCC, Pearson’s correlation coefficient; MAE, mean absolute error; MSE, mean squared error; XGBoost, extreme gradient boosting; LightGBM, light gradient boosting machine; GBDT, gradient boosting decision tree; ANN, artificial neural network.

**Figure 5:**
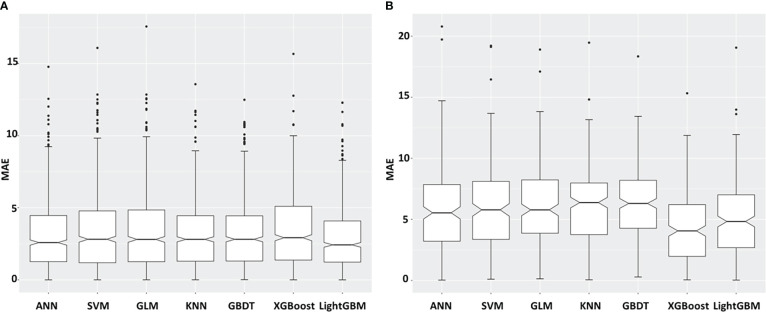
Mean absolute prediction errors (MAE) broken down for the seven different prediction models and different age groups: **(A)** 20–35 **(B)** >35 years.

As 35 years is the boundary age of childbearing, here, we divided the datasets into two different age groups (20–35 and >35 years) and analyzed the mean prediction errors by age groups. **Figures 5A, B** show absolute prediction errors in different age groups under the seven models. The LightGBM model obtained the lowest MAE value of 2.88 in the 20–35 age group than other methods. As there were 778 women under 35 years old (778/1,020, 76.30%), the LightGBM model could distinguish 774 (99.49%) women from them, and only 4 (0.51%) women were left. In addition, while the XGBoost model obtained the lowest MAE value of 4.20 years in the >35 years age group, the LightGBM model obtained the second lowest MAE value of 5.12.

## Discussion

In this study, we collected data on clinical, biochemical, and basic ultrasonographic features in a population of healthy women with the aim of constructing a quantitative system for ovarian reserve. We compared different machine learning models with respect to their prediction accuracy and stability in order to find a better one to reflect the ovarian reserve status.

In recent years, mathematical methods have been used by researchers to evaluate ovarian reserves. Younis et al. developed a multivariable scoring system, combining biochemical tests, imaging measures, and BMI to assess ovarian reserve and pregnancy rate (21). Xu and colleagues developed two models to evaluate ovarian reserve, clinical pregnancy rate, and live-birth rate (18, 19). Although these models are simple and easy to use, they are only used for infertile patients who require fertility treatments and *in vitro* fertilization (IVF) cycles, which means that they do not adequately reflect the majority of women of childbearing age. Additionally, the output result from these models is categorized, which makes it impossible to quantify ovarian function. Even though they could evaluate the reproductive prognosis, it would be challenging for these models to forecast the timing of menopause. As a result, Roberta’s study attempted to measure and describe ovarian function using the quantitative variable OvAge, a numeric variable that accurately reflects ovarian reserve in terms of both reproductive potential and time to menopause (20). They employed a single generalized linear model method since their study was the first to utilize a multi-factor model to assess and quantify ovarian age, and other characteristics like BMI that affect ovarian reserve should also be included in the model (31). Meanwhile, there were many ultrasonic measurement indicators in the model, for which special hardware was needed, and the subjective judgment of different ultrasound staff might result in an artificial mistake. In contrast to their study, we developed assessment models using a variety of machine learning methods and straightforward, objective indicators. Furthermore, seven machine learning models were constructed and analyzed to choose the most effective model for ovarian reserve quantification.

In our study, we first calculated the PCC value between different indicators and age. AFC value and AMH obtained the highest absolute PCC value, which is in accordance with the studies that said that AFC and AMH were the two most important single tests in evaluating ovarian reserve. The PCC value between AMH and AFC was as high as 0.67, indicating the effect of AMH on the stage of pre-antral and small antral follicles (32). We also revealed the AFC and AMH distributions, referring to age, and obtained fitting curves. With the prevailing age-specific reference values obtained for AMH levels based on samples from an American population in 2011, age-specific AMH reference values for Chinese women are needed (33). Our age-specific AMH distribution curve here is also similar to that of a Japanese study revealing an age-specific AMH reference for Japanese women to evaluate reproductive potential (34).

We used the assumption that ovarian age corresponds to chronological age in healthy women to investigate this novel variable of ovarian reserve. The key findings of this research are that clinical variables, blood biomarkers, and ultrasonographic characteristics may all be used to estimate ovarian reserve. After ranking analysis, including PCC, MAE, and MSE values, we determined the LightGBM model to be the best appropriate model of the seven prediction models we constructed. The LightGBM approach, which was developed to be dispersed and effective with the benefits of faster training speeds, more efficiency, and better accuracy, utilized histogram-based algorithms. In our study, the performance of the LightGBM model, which had the second-best PCC value of all the models, obtained PCC values of 0.82, 0.56, and 0.70 for the training set, the test set, and the entire dataset, respectively. MAE measures the exact differences between ovarian age and predicted ovarian age, and the LightGBM model obtained the lowest MAE value, indicating better accuracy. Moreover, the MSE value of the LightGBM model was the lowest, which showed better stability in this method. Other models, such as XGBoost and ANN, also exhibited good performance on prediction accuracy but did not perform as well in terms of stability. As a previous study used the GLM method to construct a predictive system for ovarian reserve evaluation, the results here showed that the predictive power of this method was lower than that of other methods (20). Considering that a model combining markers would not be superior to a model with a single marker, we found that the PCC values of the seven models were all higher than those of single markers, such as AMH (−0.45) and AFC (−0.43), which indicated that machine learning methods may lessen the influence of correlated markers in combined ovarian reserve marker models (35, 36).

Further, we performed an age-stratified analysis, and we found that the LightGBM model was the most suitable model for women under the age of 35, with the lowest MAE value of 2.88 years. This model could distinguish the ovarian age for women under 35 years old with an accuracy of 99.49%. As we know, 35 years is the boundary for childbearing age, and the model has the potential to be used for evaluating reproductive function and guiding childbearing (37).

Our study has several strengths. First, we assessed and quantified ovarian reserve in terms of ovarian age in a way that could be easily implemented in the clinic. For example, as the recognized natural menopause age is around 51, it is easy to evaluate the distance to an individual’s menopause (38). Further, as 35 years is the boundary for childbearing age, it is easy to predict ovarian age and compare it to this boundary, then design individual reproductive plans (37). Second, the data included in this study came from multi-centers, which covered several geographical regions of China; this made the study population more representative and improved the credibility of the results. Third, we compared the performances of seven models and selected the most effective one. Our result may be more reasonable than the former study, which used only one method.

This study has several limitations. First, our model regards ovarian age as chronological age in healthy women, which would need more strict inclusion criteria for the population. Second, due to incomplete information, limited features were used in this study. As ovarian aging is associated with additional features including lifestyle and genetic factors, these features should also be incorporated into future studies (3, 4, 39). Third, this is a cross-sectional study involving healthy population data; an external validation test should be conducted in polycystic ovarian syndrome and diminished ovarian reserve patients. A longitudinal follow-up study should be performed to assess the predicting ability. Additionally, though this is a nationwide study, the sample size from some centers was too small, which could potentially cause bias. More samples are needed to further test the model and explore more clinical applications.

## Conclusion

Taken together, machine learning methods combining multi-features, including simple and easily obtained clinical, biochemical, and ultrasonographic parameters were reliable in quantifying ovarian reserve and were better than a single indicator, providing another possible measurement to reflect ovarian reserve accurately and predict the aging degree of female ovaries individually. After comparison, the LightGBM method revealed itself to be the approach with the best quantitative effect and stability, especially in the specific age group of 20 to 35 years. In the future, this model should be tested and improved on a larger cohort.

## Data availability statement

The raw data supporting the conclusions of this article will be made available by the authors, without undue reservation.

## Ethics statement

The studies involving human participants were reviewed and approved by Tongji Hospital, Tongji Medical College, Huazhong University of Science and Technology. The patients/participants provided their written informed consent to participate in this study.

## Author contributions

SW and YL conceived and designed the study. TD and TW collected the data and developed the analytic pipeline. WR, YH, and WM led the analysis, generated the tables and figures, and wrote the manuscript. MW and FF verified and processed the underlying data. All authors contributed to the article and approved the submitted version.

## Supplementary material

Supplementary Figure 1 Coefficient values for features in different steps of LASSO regression.

## References

[1] Park SU Walsh L Berkowitz KM. Mechanisms of ovarian aging. Reprod Camb Engl (2021) 162:R19–33. doi: 10.1530/REP-21-0022 PMC9354567 33999842

[2] Practice Committee of the American Society for Reproductive Medicine. Testing and interpreting measures of ovarian reserve: a committee opinion. Fertil Steril (2020) 114:1151–7. doi: 10.1016/j.fertnstert.2020.09.134 33280722

[3] Hipp HS Charen KH Spencer JB Allen EG Sherman SL. Reproductive and gynecologic care of women with fragile X primary ovarian insufficiency (FXPOI). Menopause NYN (2016) 23:993–9. doi: 10.1097/GME.0000000000000658 PMC4998843 27552334

[4] He C Murabito JM. Genome-wide association studies of age at menarche and age at natural menopause. Mol Cell Endocrinol (2014) 382:767–79. doi: 10.1016/j.mce.2012.05.003 22613007

[5] Rizzo A Roscino MT Binetti F Sciorsci RL. Roles of reactive oxygen species in female reproduction. Reprod Domest Anim Zuchthyg (2012) 47:344–52. doi: 10.1111/j.1439-0531.2011.01891.x 22022825

[6] Duncan FE Gerton JL. Mammalian oogenesis and female reproductive aging. Aging (2018) 10:162–3. doi: 10.18632/aging.101381 PMC5842846 29410392

[7] Briley SM Jasti S McCracken JM Hornick JE Fegley B Pritchard MT. Reproductive age-associated fibrosis in the stroma of the mammalian ovary. Reprod Camb Engl (2016) 152:245–60. doi: 10.1530/REP-16-0129 PMC4979755 27491879

[8] Foley KG Pritchard MT Duncan FE. Macrophage-derived multinucleated giant cells: hallmarks of the aging ovary. Reprod Camb Engl (2021) 161:V5–9. doi: 10.1530/REP-20-0489 PMC7856073 33258461

[9] de Vet A Laven JSE de Jong FH Themmen APN Fauser BCJM. Antimüllerian hormone serum levels: A putative marker for ovarian aging. Fertil Steril (2002) 77:357–62. doi: 10.1016/s0015-0282(01)02993-4 11821097

[10] Steiner AZ Pritchard D Stanczyk FZ Kesner JS Meadows JW Herring AH. Association between biomarkers of ovarian reserve and infertility among older women of reproductive age. JAMA (2017) 318:1367–76. doi: 10.1001/jama.2017.14588 PMC5744252 29049585

[11] Zarek SM Mitchell EM Sjaarda LA Mumford SL Silver RM Stanford JB. Antimüllerian hormone and pregnancy loss from the effects of aspirin in gestation and reproduction trial. Fertil Steril (2016) 105:946–952.e2. doi: 10.1016/j.fertnstert.2015.12.003 26707905 PMC4821799

[12] Van Calster B Wynants L. Machine learning in medicine. N Engl J Med (2019) 380:2588. doi: 10.1056/NEJMc1906060 31242379

[13] Ngiam KY Khor IW. Big data and machine learning algorithms for health-care delivery. Lancet Oncol (2019) 20:e262–73. doi: 10.1016/S1470-2045(19)30149-4 31044724

[14] Waljee AK Higgins PDR. Machine learning in medicine: A primer for physicians. Am J Gastroenterol (2010) 105:1224–6. doi: 10.1038/ajg.2010.173 20523307

[15] Blank C Wildeboer RR DeCroo I Tilleman K Weyers B de Sutter P. Prediction of implantation after blastocyst transfer in in vitro fertilization: A machine-learning perspective. Fertil Steril (2019) 111:318–26. doi: 10.1016/j.fertnstert.2018.10.030 30611557

[16] Liao S Pan W Dai W-Q Jin L Huang G Wang R. Development of a dynamic diagnosis grading system for infertility using machine learning. JAMA Netw Open (2020) 3:e2023654. doi: 10.1001/jamanetworkopen.2020.23654 33165608 PMC7653500

[17] Qiu J Li P Dong M Xin X Tan J. Personalized prediction of live birth prior to the first *in vitro* fertilization treatment: A machine learning method. J Transl Med (2019) 17:317. doi: 10.1186/s12967-019-2062-5 31547822 PMC6757430

[18] Xu H Feng G Wang H Han Y Yang R Song Y. A novel mathematical model of true ovarian reserve assessment based on predicted probability of poor ovarian response: A retrospective cohort study. J Assist Reprod Genet (2020) 37:963–72. doi: 10.1007/s10815-020-01700-1 PMC7183040 32318905

[19] Xu H Shi L Feng G Xiao Z Chen L Li R. An ovarian reserve assessment model based on anti-müllerian hormone levels, follicle-stimulating hormone levels, and age: Retrospective cohort study. J Med Internet Res (2020) 22:e19096. doi: 10.2196/19096 32667898 PMC7546624

[20] Venturella R Lico D Sarica A Falbo MP Gulletta E Morelli M. OvAge: A new methodology to quantify ovarian reserve combining clinical, biochemical and 3D-ultrasonographic parameters. J Ovarian Res (2015) 8:21. doi: 10.1186/s13048-015-0149-z 25881987 PMC4392473

[21] Younis JS Jadaon J Izhaki I Haddad S Radin O Bar-Ami S. A simple multivariate score could predict ovarian reserve, as well as pregnancy rate, in infertile women. Fertil Steril (2010) 94:655–61. doi: 10.1016/j.fertnstert.2009.03.036 19368907

[22] Zhang H Wang J Sun Z Zurada JM Pal NR. Feature selection for neural networks using group lasso regularization. IEEE Trans Knowl Data Eng (2020) 32:659–73. doi: 10.1109/TKDE.2019.2893266

[23] Mishra M Srivastava M. A view of artificial neural network. (IEEE.) (2014). pp. 1–3. doi: 10.1109/ICAETR.2014.7012785.

[24] Basak D Srimanta P Patranbis DC. Support vector regression. Neural Inf Process Lett Rev (2007) 11. doi: 10.1007/978-1-4302-5990-9_4

[25] Friedman J. Greedy function approximation: A gradient boosting machine. Ann Stat (2001) 29:1189–232. doi: 10.2307/2699986

[26] Zhang Z. Introduction to machine learning: K-nearest neighbors. Ann Transl Med (2016) 4:218–8. doi: 10.21037/atm.2016.03.37 PMC4916348 27386492

[27] Nwanganga F Chapple M. K-nearest neighbors. (Wiley) (2020). pp. 221–49. doi: 10.1002/9781119591542.ch6.

[28] Chen T Guestrin C. XGBoost: A scalable tree boosting system. In: Proceedings of the 22nd ACM SIGKDD international conference on knowledge discovery and data mining. San Francisco California USA: ACM (2016). p. 785–94. doi: 10.1145/2939672.2939785

[29] Meng Q. LightGBM: A highly efficient gradient boosting decision tree. Neural Information Processing Systems Curran Associates Inc. (2017) 3149–3157.

[30] Xiao J Ding R Xu X Guan H Feng X Sun T. Comparison and development of machine learning tools in the prediction of chronic kidney disease progression. J Transl Med (2019) 17:119. doi: 10.1186/s12967-019-1860-0 30971285 PMC6458616

[31] >Sermondade N Huberlant S Bourhis-Lefebvre V Arbo E Gallot V Colombani M. Female obesity is negatively associated with live birth rate following IVF: A systematic review and meta-analysis. Hum Reprod Update (2019) 25:439–51. doi: 10.1093/humupd/dmz011 30941397

[32] Traversari J Aepli H Knutti B Lüttgenau J Bruckmaier RM Bollwein H. Relationships between antral follicle count, blood serum concentration of anti-müllerian hormone and fertility in mares. Schweiz Arch Tierheilkd (2019) 161:627–38. doi: 10.17236/sat00225 31586925

[33] Seifer DB Baker VL Leader B. Age-specific serum anti-müllerian hormone values for 17,120 women presenting to fertility centers within the united states. Fertil Steril (2011) 95:747–50. doi: 10.1016/j.fertnstert.2010.10.011 21074758

[34] Segawa T Omi K Watanabe Y Sone Y Handa M Kuroda M. Age-specific values of access anti-müllerian hormone immunoassay carried out on Japanese patients with infertility: A retrospective large-scale study. BMC Womens Health (2019) 19:57. doi: 10.1186/s12905-019-0752-z 31023297 PMC6485128

[35] Lorusso F Vicino M Lamanna G Trerotoli P Serio G Depalo R. Performance of different ovarian reserve markers for predicting the numbers of oocytes retrieved and mature oocytes. Maturitas (2007) 56:429–35. doi: 10.1016/j.maturitas.2006.11.007 17184937

[36] Broer SL van Disseldorp J Broeze KA Dolleman M Opmeer BC Bossuyt P. Added value of ovarian reserve testing on patient characteristics in the prediction of ovarian response and ongoing pregnancy: an individual patient data approach. Hum Reprod Update (2013) 19:26–36. doi: 10.1093/humupd/dms041 23188168

[37] West C. Age and infertility. Br Med J Clin Res Ed (1987) 294:853–4. doi: 10.1136/bmj.294.6576.853 PMC1245918 3105771

[38] Zhu D Chung H-F Dobson AJ Pandeya N Giles GG Bruinsma F. Age at natural menopause and risk of incident cardiovascular disease: A pooled analysis of individual patient data. Lancet Public Health (2019) 4:e553–64. doi: 10.1016/S2468-2667(19)30155-0 PMC7118366 31588031

[39] Laisk T Tšuiko O Jatsenko T Hõrak P Otala M Lahdenperä M. Demographic and evolutionary trends in ovarian function and aging. Hum Reprod Update (2019) 25:34–50. doi: 10.1093/humupd/dmy031 30346539

