# Supplementary figures and images for: Assessment and quantification of ovarian reserve on the basis of machine learning models
Source: Front Endocrinol (Lausanne). 2023 Mar 15;14:1087429. doi: 10.3389/fendo.2023.1087429 (PMC10050589; doi:10.3389/fendo.2023.1087429)

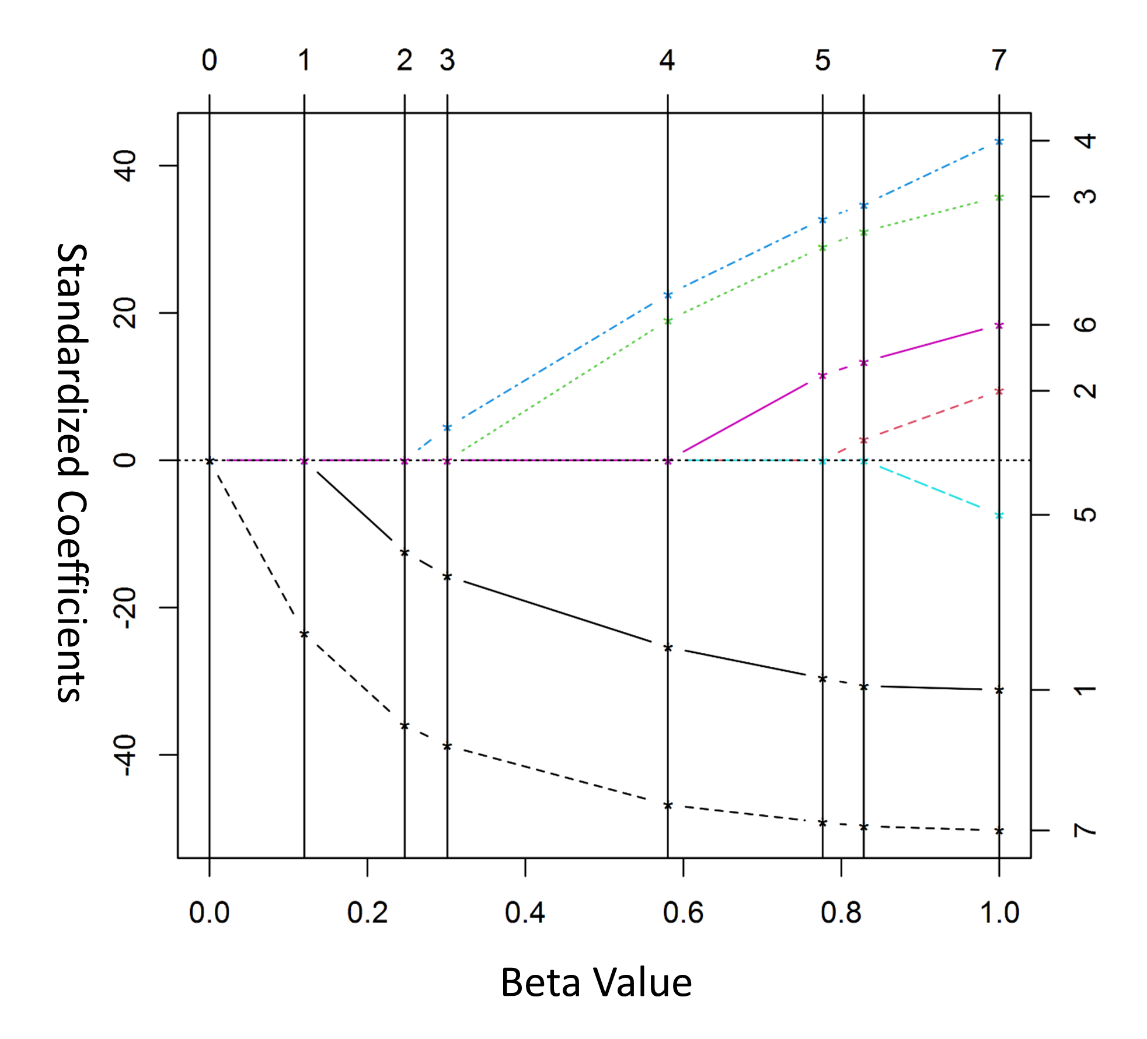

Supplement: Supplementary Figure 1 — Coefficient values for features in different steps of LASSO regression. [file Image_1.tif]
