# Supplementary material for: Assessment and quantification of ovarian reserve on the basis of machine learning models
Source: Front Endocrinol (Lausanne). 2023 Mar 15;14:1087429. doi: 10.3389/fendo.2023.1087429 (PMC10050589; doi:10.3389/fendo.2023.1087429)
Supplement: Supplementary file 2 [file Table_1.docx]

| Model | R package/function | Parameter |
| --- | --- | --- |
| ANN | neuralnet/neuralnet | hidden=c(5,2), linear.output=T |
| SVM | e1071/svm | kernel = "linear" |
| GLM | glm |  |
| KNN | kknn/train.kknn | k = 22 |
| GBDT | gbm/gbm | n.trees = 252, shrinkage = 0.01 |
| XGBoost | xgboost/xgb.train | nrounds = 5, booster="gbtree", nfold=3 |
| LightGBM | lightgbm/lgb.train | nrounds = 500, early_stopping_rounds = 40 |

Table S1. Parameters used in the machine learning models.
