# Supplementary material for: Assessment and quantification of ovarian reserve on the basis of machine learning models
Source: Front Endocrinol (Lausanne). 2023 Mar 15;14:1087429. doi: 10.3389/fendo.2023.1087429 (PMC10050589; doi:10.3389/fendo.2023.1087429)
Supplement: Supplementary file 3 [file Table_2.docx]

**Table S2.** CP value for different Steps in LASSO regression.

| **Step** | **CP** |
| --- | --- |
| 0 | 469.816 |
| 1 | 321.172 |
| 2 | 208.819 |
| 3 | 173.594 |
| 4 | 48.231 |
| 5 | 14.097 |
| 6 | 11.828 |
| 7 | 8.000 |
